# Prevention of Neural Tube Defects: A Cross-Sectional Study of the Uptake of Folic Acid Supplementation in Nearly Half a Million Women

**DOI:** 10.1371/journal.pone.0089354

**Published:** 2014-02-19

**Authors:** Jonathan P. Bestwick, Wayne J. Huttly, Joan K. Morris, Nicholas J. Wald

**Affiliations:** Wolfson Institute of Preventive Medicine, Barts and the London School of Medicine and Dentistry, Queen Mary University of London, London, United Kingdom; University of Missouri, United States of America

## Abstract

**Background:**

Taking folic acid supplements before pregnancy to reduce the risk of a neural tube defect (NTD) is especially important in countries without universal folic acid fortification. The extent of folic acid supplementation among women who had antenatal screening for Down’s syndrome and NTDs at the Wolfson Institute of Preventive Medicine, London between 1999 and 2012 was assessed.

**Methods and Findings:**

466,860 women screened provided details on folic acid supplementation. The proportion of women who took folic acid supplements before pregnancy was determined according to year and characteristics of the women. The proportion of women taking folic acid supplements before pregnancy declined from 35% (95% CI 34%–35%) in 1999–2001 to 31% (30%–31%) in 2011–2012. 6% (5%–6%) of women aged under 20 took folic acid supplements before pregnancy compared with 40% of women aged between 35 and 39. Non-Caucasian women were less likely to take folic acid supplements before pregnancy than Caucasian women; Afro-Caribbean 17% (16%–17%), Oriental 25% (24%–25%) and South Asian 20% (20%–21%) compared with 35% (35%–35%) for Caucasian women. 51% (48%–55%) of women who previously had an NTD pregnancy took folic acid supplements before the current pregnancy.

**Conclusions:**

The policy of folic acid supplementation is failing and has led to health inequalities. This study demonstrates the need to fortify flour and other cereal grain with folic acid in all countries of the world.

## Introduction

In 1991 the results of the Medical Research Council Vitamin Study were published. This randomised controlled trial showed that taking folic acid before conception reduced the risk of a neural tube defect (NTD) pregnancy by an estimated 72% [1]. A high enough daily intake of folate to provide such a reduction in risk cannot easily be obtained by dietary changes so supplementation is necessary, particularly in countries where folic acid fortification has not been implemented. Mandatory fortification has been introduced in over 70 countries including the US, Canada and Australia [2], but despite evidence that such fortification is effective [3]–[5], over 120 countries have not introduced mandatory folic acid fortification, including all countries in the European Union, so women of child bearing age need to take folic acid supplements before pregnancy to reduce their risk of an NTD pregnancy. In 1998, 6 years after the UK Department of Health recommendation on the intake of folic acid before pregnancy [6] the proportion of women from one hospital screened at the Wolfson Institute of Preventive Medicine, London who took folic acid supplements before pregnancy was reported to be 42% (523/1238) [7]. We here assess the extent of folic acid supplementation using data from almost half a million women screened at the Wolfson Institute between 1999 and 2012.

## Methods

### Ethics Statement

The results in the paper arose out of an audit of our screening service for which no explicit consent was sought but women screened were informed that data arising from the screening programme might be used to make improvements to the services provided. All the data were anonymous and no enquiries or procedures were performed other than those that formed part of the routine service so ethical approval was not required.

In 1998 the antenatal Down’s syndrome and NTD screening service at the Wolfson Institute of Preventive Medicine, London introduced a question on screening request forms asking whether women had (i) started taking folic acid supplements before pregnancy, (ii) started once pregnancy was confirmed or (iii) not taken folic acid supplements. Between January 1999 and December 2012 520,570 pregnant women were routinely screened at the Wolfson Institute for Down’s syndrome using the late first trimester (11–13 weeks’ gestation) Combined test (61%; median gestational age 12 weeks, 5 days) or the early second trimester (15–20 weeks’ gestation) Quadruple test (39%; median gestational age 16 weeks, 3 days). Women screened with the Quadruple test were also screened for NTDs using maternal serum alphafetoprotein, collected as part of the Quadruple test, and a further 867 women were screened for NTDs only. A total of 466,860 (90%; lower proportion in earlier years as the new request forms were being introduced) provided data on folic acid supplement status. The proportion of women who started taking folic acid supplements before pregnancy was determined for each calendar year. Data on the following specified factors that are routinely collected as part of the screening service were used to determine the independent effects of each: previous NTD pregnancy (yes/no), previous Down’s syndrome pregnancy (yes/no), ethnicity (Caucasian, Afro-Caribbean, South Asian [Indian, Pakistani, Bangladeshi or Sri-Lankan], Oriental [Chinese, Japanese or South East Asian] or Other), insulin dependent diabetes mellitus (yes/no), in-vitro fertilisation (IVF, yes/no), smoker (yes/no), maternal weight (five 10 kg categories) and maternal age (seven 5-year categories), region of residence (categorised as strategic health authorities and the Isle of Man), and time of screening (first or second trimester). The relative use of taking folic acid supplements before pregnancy versus not taking folic acid supplements before pregnancy was calculated according to these factors and categories. Adjusted relative use estimates were calculated using multivariate Poisson regression with robust variances [8]. The average marginal effects were calculated to estimate the adjusted percentages of women taking folic acid before pregnancy i.e. the percentages independent of the specified factors. All analyses were performed in Stata version 12 (StatCorp, College Station, Texas).

## Results

**Figure 1:**
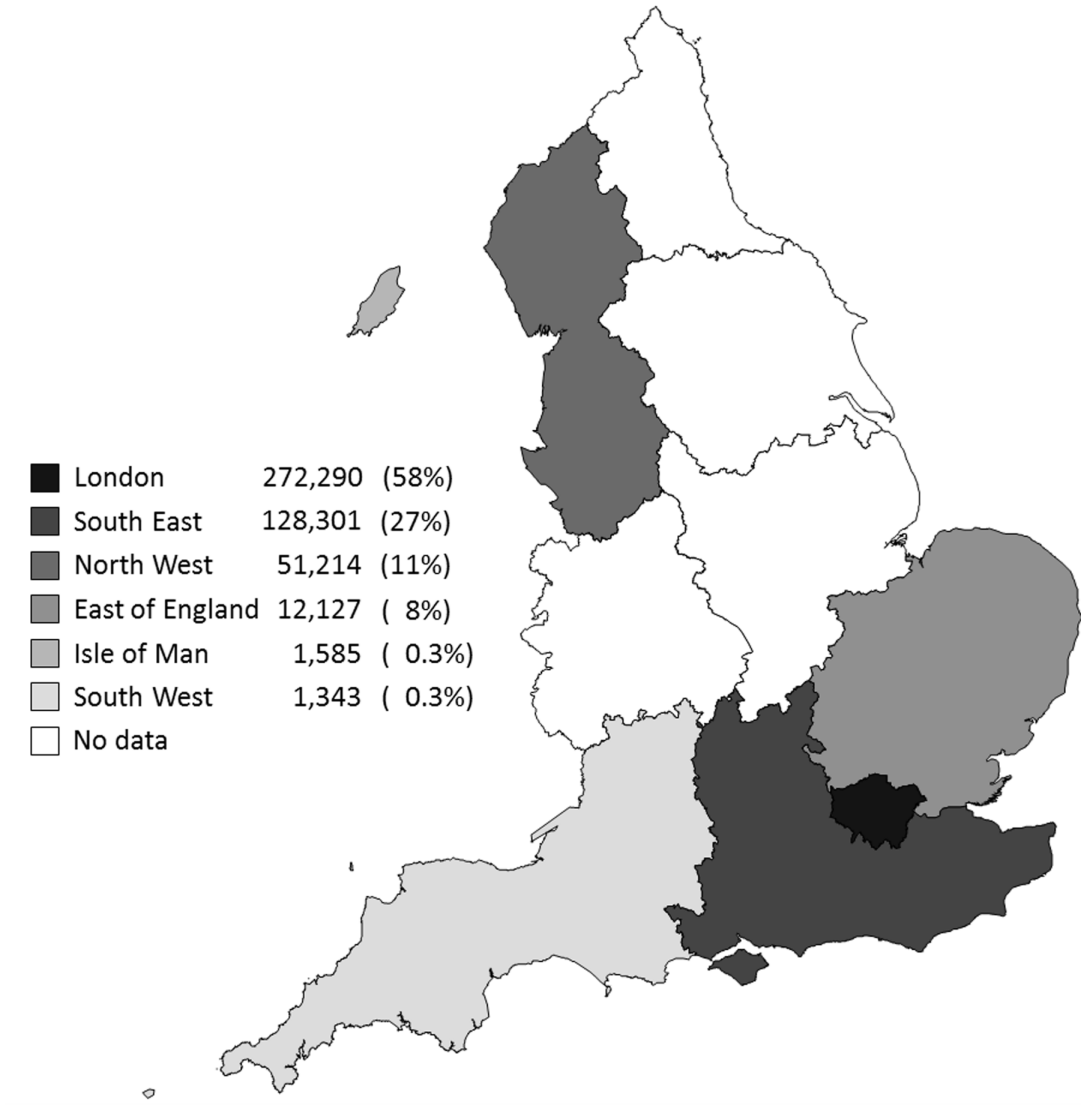
Number of women who provided folic acid supplement status according to strategic health authority and the Isle of Man.

Figure 1 shows the strategic health authorities of England, and the Isle of Man where women provided blood samples for screening. Fifty-eight percent of women were from London, 27% from the South East, 11% from the North West, with the remainder from the East of England, Isle of Man and the South West.

**Figure 2:**
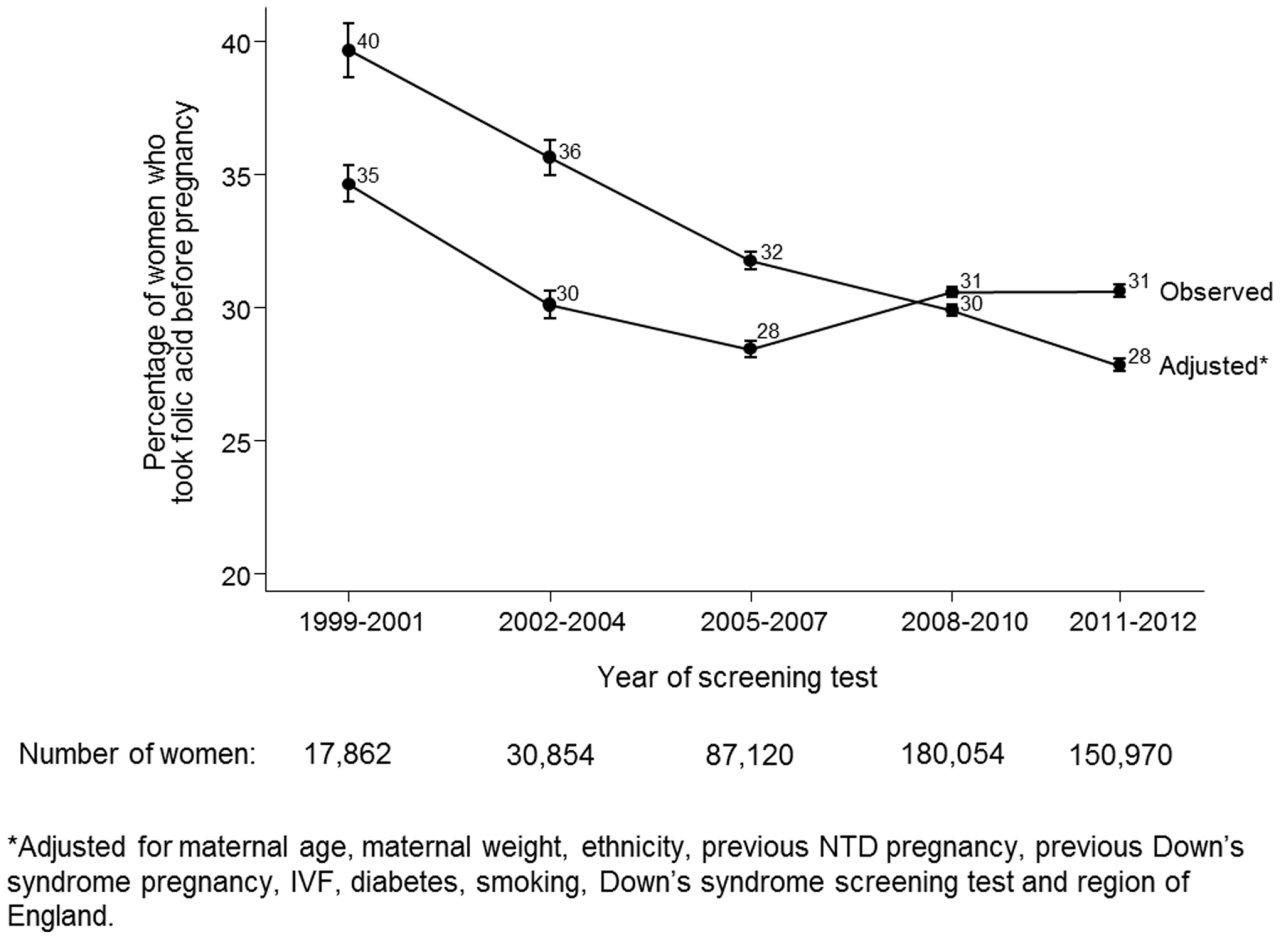
Observed and adjusted* percentage of women taking folic acid supplements before pregnancy according to year.

All factors were statistically significantly associated with taking folic acid supplements before pregnancy (all p<0.001) and each factor, adjusting for all other factors, remained statistically significant (all p<0.001; see Table S1). Figure 2 shows that between 1999–2001 and 2002–2004 the observed proportion of women taking folic acid supplements before pregnancy reduced from 35% (95% CI 34%–35%) to 31% (30%–31%) and in the following ten years the proportion remained stable. The adjusted percentage taking folic acid supplements before pregnancy reduced in each period from 40% (39%–41%) in 1999–2001 to 28% (28%–28%) in 2011–2012.

**Figure 3:**
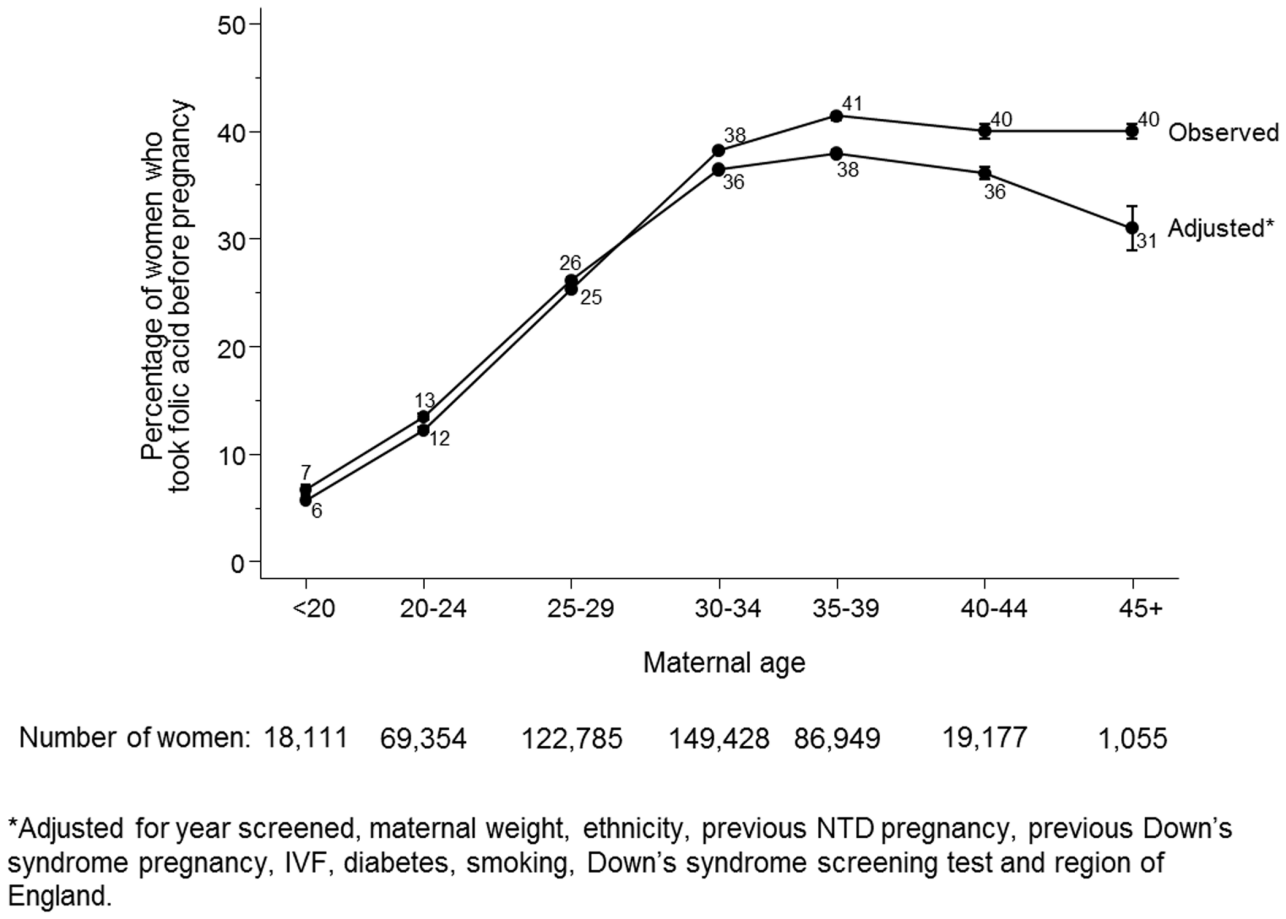
Observed and adjusted* percentage of women taking folic acid supplements before pregnancy according to maternal age.

Figure 3 shows that younger women were less likely to take folic acid supplements before pregnancy than older women; 6% (5%–6%) of women aged under 20 and 40% of women aged between 35 and 39. The adjusted percentage of women taking folic acid supplements before pregnancy peaked at 38% (38%–38%) in women aged 35–39 and was 31% (29%–33%) in women aged 45 or older.

**Figure 4:**
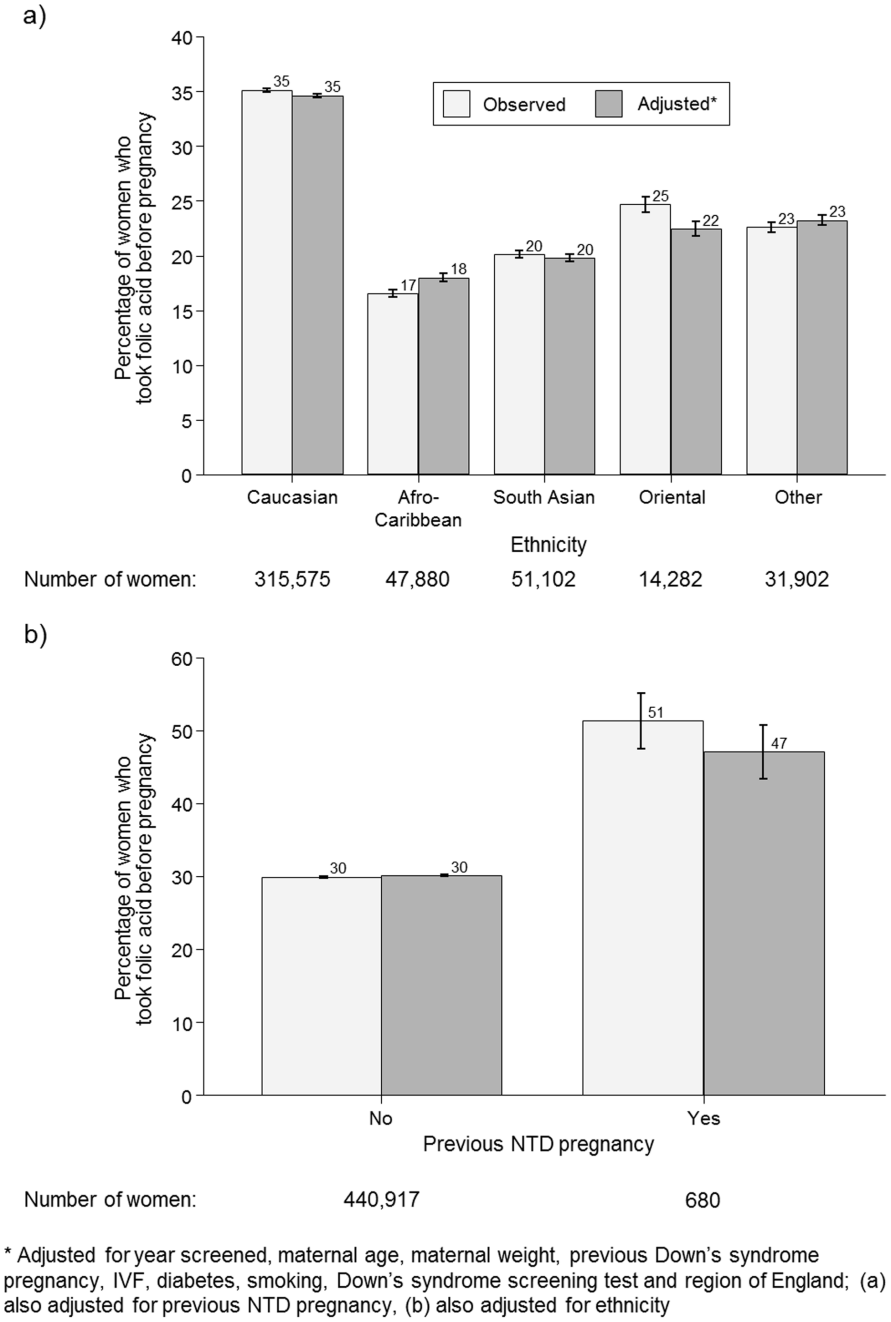
Observed and adjusted* percentage of women taking folic acid supplements before pregnancy according to ethnicity (a) and previous neural tube defect (NTD) pregnancy (b).

Figure 4a shows that non-Caucasian women were less likely to take folic acid supplements before pregnancy than Caucasian women; 35% (35%–35%) of Caucasian women took folic acid supplements before pregnancy compared with 17% (16%–17%) of Afro-Caribbean women, 20% (20%–21%) of South Asian women, 25% (24%–25%) of Oriental women and 23% (22%–23%) of women of other ethnic groups. Adjusted percentage estimates were similar to the unadjusted values.

Figure 4b shows that 51% (48%–55%) of women with a previous NTD affected pregnancy took folic acid supplements before their current pregnancy compared with 30% (30%–30%) of women without a previous NTD affected pregnancy (adjusted figures similar).

Table S1 shows results for other factors and gives unadjusted and adjusted relative use of folic acid supplements before pregnancy, from which adjusted percentages were derived. These results show that (i) women who had undergone IVF were most likely to take folic acid supplements prior to pregnancy, but despite being under close medical supervision 20% of them were not doing so, (ii) women who had previously had a pregnancy affected with Down’s syndrome were more likely to take folic acid supplements before pregnancy than those who had not, but the proportion was still only about half, (iii) insulin dependent diabetic women were more likely to take folic acid supplements before pregnancy than non-insulin dependent diabetic women, (iv) smokers were less likely to take folic acid supplements before pregnancy than non-smokers and (v) women who were screened in the second trimester were less likely to take folic acid supplements than women screened in the first trimester, possibly because a greater proportion of women who had second trimester screening had unplanned pregnancies that were confirmed later in gestation.

## Discussion

This study has four main findings. Firstly, relatively few women are taking folic acid supplements before pregnancy, with the proportion declining over the study period, from 35% in 1991–2001 to about 30% in 2011–2012. However, as women are having children at older ages and more women are having IVF, and these women were more likely to take folic acid supplements before pregnancy, the extent of the year-specific downwards trend is concealed. Perhaps more relevant is the observation that the adjusted percentage of women taking folic acid supplements before pregnancy declined from 40% in 1999–2001 to 28% in 2011–2012. Secondly non-Caucasian women were about half as likely as Caucasian women to take folic acid supplements before pregnancy. This probably reflects socio-economic status, demonstrating an example of health inequalities. Thirdly, whilst women who had previously had an NTD pregnancy were more likely to take folic acid supplements before pregnancy than women who had not, only about half of them did. Further, 16 women had had two previous NTD pregnancies; only five of them took folic acid supplements before pregnancy. Fourthly, younger women were far less likely to take folic acid supplements before pregnancy; women younger than 20 were five times less likely to take folic acid supplements before pregnancy than women aged 30 or over. This may be partly due to more unplanned pregnancies and less knowledge of the benefits of folic acid supplementation in younger women. After adjustment the percentage of women aged 45 or older taking folic acid supplements prior to pregnancy was 31%, lower than the percentage of women aged 40–44 (36%) despite the unadjusted percentages being the same (40%). The main contributor to the lower adjusted percentage for women aged 45 or older compared with women aged 40–44 is the proportion of women who had undergone IVF treatment (who were most likely to take folic acid before pregnancy) in the 45 or older age group; 34% compared with 7% of women aged 40–44.

During the study period the proportion of women who started taking folic acid supplements after pregnancy had been confirmed increased from 45% to 62% (see Table S1). However, to prevent NTDs folic acid supplements need to be taken before pregnancy; there is little to no benefit of taking folic acid supplements after pregnancy has been confirmed as the neural tube (and any defect) will have formed by around 8 weeks of pregnancy.

A strength of this study is the large sample size; almost half a million pregnancies resulting in very narrow confidence intervals of our estimates and even small differences are highly statistically significant. The results show that the policy of folic acid supplementation has failed. The policy exacerbates health inequalities among ethnic minorities and younger women. Our study is based on women from England and the Isle of Man, but the results are similar to other countries; a review of 34 studies conducted in 8 countries between 1992 and 2001 showed that nowhere was the proportion of women taking folic acid supplements before pregnancy greater than 50% and that overall it was about 25% [9]. Our results show that over the past decade the proportion has decreased in England. A weakness of this study is that it is limited to women that had antenatal screening for Down’s syndrome and neural tube defects. Uptake of screening in England and Wales was 53% in 2007, increasing to 74% in 2011 [10].

The global number of spina bifida and anencephaly births has been estimated at 300,000 to 400,000 per year [11]. In the 20 years since the publication of the MRC Vitamin Study 1,800,000 cases could have been prevented on the basis that fortification alone would prevent one-third of cases. Among women that took folic acid supplements this would be even higher. In contrast, an estimated worldwide total of 10,000 people were disabled from thalidomide [12] and this led to transformation of the drug regulatory system. It is a public health tragedy that in spite of the fortification initiative in many countries most have not introduced mandatory folic acid fortification [2].

It is widely judged that folic acid fortification and supplementation is safe. Concerns over the possible increased risk of cancer being associated with folic acid fortification are not justified [13], [14] and a large meta-analysis of placebo-controlled randomised trials of folic acid (median daily dose 2.0 mg over an average 5 years of follow-up) showed no increase in cancer incidence in those taking folic acid [15]. Moreover there is evidence that the generally recommended daily dose of folic acid supplementation is too low at 0.4 mg; about 80% of all NTDs could be prevented if it were 5 mg instead of about 50% with 0.4 mg [16]. Therefore fortification would offer additional protection against NTDs for women taking the current recommended daily dose.

The prevention of neural tube defects is a global problem. Our results show that public health policy cannot rely on pre-pregnancy folic acid supplementation alone. Improved education in communities with particularly low rates of pre-pregnancy folic acid supplementation may increase the rates but for all women to benefit countries that have not introduced folic acid fortification should do so to avert a preventable serious birth defect responsible for still births, severe physical disability and needless therapeutic abortions.

## Supporting Information

Table S1 Percentage of women taking folic acid before pregnancy, once pregnancy confirmed or not at all with unadjusted and adjusted relative use compared with reference categories.
(DOCX)
