# Supplementary material for: Prevention of Neural Tube Defects: A Cross-Sectional Study of the Uptake of Folic Acid Supplementation in Nearly Half a Million Women
Source: PLoS One. 2014 Feb 19;9(2):e89354. doi: 10.1371/journal.pone.0089354 (PMC3929694; doi:10.1371/journal.pone.0089354)
Supplement: Table S1 — Percentage of women taking folic acid before pregnancy, once pregnancy confirmed or not at all with unadjusted and adjusted relative use compared with reference categories. (DOCX) [file pone.0089354.s001.docx]

Table S1: Percentage of women taking folic acid before pregnancy, once pregnancy confirmed or not at all with unadjusted and adjusted relative use compared with reference categories.

|  |  | **Folic acid supplement use (%)** | | |  | **Relative use of folic acid supplements before pregnancy compared with reference category (95% CI)** | | **Adjusted* folic acid supplement use before pregnancy (%, 95% CI)** |
| --- | --- | --- | --- | --- | --- | --- | --- | --- |
|  | **Number of women** | **Before pregnancy (95% CI)** | **After pregnancy confirmed** | **Not at all** |  |  |  |  |
|  |  |  |  |  |  |  |  |  |
|  |  |  |  |  |  | **Unadjusted** | **Adjusted*** |  |
| **Year screened** |  |  |  |  |  |  |  |  |
| 1999-2001 | 17,862 | 34.7 (34.0-35.4) | 45.4 | 19.9 |  | 1.00 (ref) | 1.00 (ref) | 39.6 (38.6-40.7) |
| 2002-2004 | 30,854 | 30.1 (29.6-30.7) | 51.0 | 18.8 |  | 0.87 (0.85-0.89) | 0.90 (0.87-0.92) | 35.6 (35.0-36.3) |
| 2005-2007 | 87,120 | 28.5 (28.2-28.8) | 52.6 | 18.9 |  | 0.82 (0.80-0.84) | 0.80 (0.78-0.82) | 31.8 (31.4-32.1) |
| 2008-2010 | 180,054 | 30.6 (30.4-30.8) | 58.3 | 11.1 |  | 0.88 (0.86-0.90) | 0.75 (0.73-0.78) | 29.9 (29.7-30.1) |
| 2011-2012 | 150,970 | 30.6 (30.4-30.9) | 61.8 | 7.6 |  | 0.88 (0.86-0.90) | 0.70 (0.68-0.72) | 27.8 (27.6-28.1) |
|  |  |  |  |  |  |  |  |  |
| **Maternal age** |  |  |  |  |  |  |  |  |
| <20 | 18,111 | 5.7 ( 5.3- 6.0) | 67.1 | 27.2 |  | 1.00 (ref) | 1.00 (ref) | 6.7 ( 6.2- 7.1) |
| 20-24 | 69,354 | 12.2 (12.0-12.5) | 68.6 | 19.2 |  | 2.15 (2.02-2.29) | 2.02 (1.89-2.16) | 13.4 (13.1-13.7) |
| 25-29 | 122,785 | 25.2 (25.0-25.5) | 62.0 | 12.8 |  | 4.45 (4.19-4.72) | 3.93 (3.68-4.19) | 26.1 (25.9-26.4) |
| 30-34 | 149,428 | 38.2 (37.9-38.4) | 52.8 | 9.0 |  | 6.72 (6.33-7.14) | 5.47 (5.13-5.84) | 36.4 (36.2-36.7) |
| 35-39 | 86,949 | 41.4 (41.1-41.7) | 49.8 | 8.8 |  | 7.29 (6.87-7.74) | 5.71 (5.35-6.09) | 38.0 (37.6-38.3) |
| 40-44 | 19,177 | 40.0 (39.3-40.7) | 49.5 | 10.6 |  | 7.04 (6.62-7.49) | 5.43 (5.08-5.80) | 36.1 (35.5-36.8) |
| ≥45 | 1,055 | 42.1 (39.1-45.1) | 44.5 | 13.5 |  | 7.41 (6.76-8.13) | 4.66 (4.25-5.10) | 31.0 (29.0-33.0) |
|  |  |  |  |  |  |  |  |  |
| **Ethnicity** |  |  |  |  |  |  |  |  |
| Caucasian | 315,575 | 35.1 (35.0-35.3) | 55.4 | 9.5 |  | 1.00 (ref) | 1.00 (ref) | 34.6 (34.5-34.8) |
| Oriental | 14,282 | 24.7 (24.0-25.4) | 59.1 | 16.2 |  | 0.70 (0.68-0.72) | 0.65 (0.63-0.67) | 22.4 (21.8-23.1) |
| Other | 31,902 | 22.6 (22.1-23.1) | 57.9 | 19.5 |  | 0.64 (0.63-0.66) | 0.67 (0.66-0.69) | 23.2 (22.8-23.7) |
| South Asian | 51,102 | 20.1 (19.8-20.5) | 64.1 | 15.8 |  | 0.57 (0.56-0.58) | 0.57 (0.56-0.58) | 19.8 (19.4-20.2) |
| Afro-Caribbean | 47,880 | 16.6 (16.2-16.9) | 63.1 | 20.3 |  | 0.47 (0.46-0.48) | 0.52 (0.51-0.53) | 18.0 (17.6-18.4) |
|  |  |  |  |  |  |  |  |  |
| **Previous neural tube defect pregnancy** |  |  |  |  |  |  |  |  |
| No | 440,917 | 29.9 (29.8-30.1) | 57.4 | 12.6 |  | 1.00 (ref) | 1.00 (ref) | 30.1 (30.0-30.2) |
| Yes | 680 | 51.3 (47.5-55.1) | 40.0 | 8.7 |  | 1.72 (1.59-1.85) | 1.57 (1.45-1.69) | 47.1 (43.4-50.8) |
|  |  |  |  |  |  |  |  |  |
| **Previous Down's syndrome pregnancy** |  |  |  |  |  |  |  |  |
| No | 463,161 | 30.3 (30.2-30.4) | 57.4 | 12.3 |  | 1.00 (ref) | 1.00 (ref) | 30.1 (30.0-30.2) |
| Yes | 585 | 54.0 (49.9-58.1) | 36.8 | 9.2 |  | 1.78 (1.65-1.92) | 1.43 (1.32-1.54) | 42.9 (39.7-46.2) |
|  |  |  |  |  |  |  |  |  |
| **In vitro fertilisation** |  |  |  |  |  |  |  |  |
| No | 452,733 | 29.4 (29.3-29.6) | 58.2 | 12.4 |  | 1.00 (ref) | 1.00 (ref) | 29.4 (29.2-29.5) |
| Yes | 7,962 | 82.9 (82.0-83.7) | 14.1 | 3.0 |  | 2.82 (2.78-2.85) | 2.09 (2.06-2.12) | 61.3 (60.6-62.1) |
|  |  |  |  |  |  |  |  |  |
| **Insulin dependent diabetes** |  |  |  |  |  |  |  |  |
| No | 460,008 | 30.4 (30.2-30.5) | 57.4 | 12.2 |  | 1.00 (ref) | 1.00 (ref) | 30.1 (30.0-30.2) |
| Yes | 1,651 | 38.0 (35.6-40.4) | 51.8 | 10.2 |  | 1.25 (1.18-1.33) | 1.24 (1.17-1.32) | 37.4 (35.1-39.7) |
|  |  |  |  |  |  |  |  |  |
| **Smoker** |  |  |  |  |  |  |  |  |
| No | 405,844 | 32.8 (32.6-32.9) | 56.3 | 10.9 |  | 1.00 (ref) | 1.00 (ref) | 31.9 (31.8-32.1) |
| Yes | 53,375 | 11.8 (11.5-12.1) | 67.2 | 21.0 |  | 0.36 (0.35-0.37) | 0.42 (0.41-0.43) | 13.5 (13.2-13.9) |
|  |  |  |  |  |  |  |  |  |
| **Maternal weight** |  |  |  |  |  |  |  |  |
| <50kg | 22,246 | 21.2 (20.7-21.8) | 61.7 | 17.1 |  | 1.00 (ref) | 1.00 (ref) | 28.7 (27.9-29.4) |
| 50-59kg | 114,768 | 29.8 (29.6-30.1) | 57.7 | 12.4 |  | 1.41 (1.37-1.45) | 1.08 (1.05-1.11) | 31.0 (30.7-31.2) |
| 60-69kg | 146,022 | 33.2 (32.9-33.4) | 55.9 | 10.9 |  | 1.56 (1.52-1.61) | 1.09 (1.06-1.12) | 31.2 (31.0-31.5) |
| 70-79kg | 87,517 | 31.9 (31.6-32.2) | 56.4 | 11.6 |  | 1.50 (1.46-1.55) | 1.05 (1.02-1.08) | 30.2 (29.9-30.5) |
| ≥80kg | 81,854 | 27.4 (27.1-27.7) | 60.0 | 12.6 |  | 1.29 (1.26-1.33) | 0.94 (0.92-0.97) | 27.1 (26.8-27.4) |
|  |  |  |  |  |  |  |  |  |
| **Strategic health authority/Isle of Man** |  |  |  |  |  |  |  |  |
| London | 272,290 | 28.1 (27.9-28.2) | 59.0 | 12.9 |  | 1.00 (ref) | 1.00 (ref) | 28.0 (27.8-28.2) |
| South East | 128,301 | 34.2 (34.0-34.5) | 53.6 | 12.1 |  | 1.22 (1.21-1.23) | 1.20 (1.18-1.21) | 33.5 (33.2-33.8) |
| North West | 51,214 | 33.7 (33.3-34.1) | 57.7 | 8.5 |  | 1.20 (1.19-1.22) | 1.24 (1.22-1.26) | 34.7 (34.2-35.1) |
| East of England | 12,127 | 25.1 (24.3-25.9) | 61.4 | 13.5 |  | 0.89 (0.87-0.92) | 1.06 (1.03-1.10) | 29.7 (28.8-30.7) |
| Isle of Man | 1,585 | 38.8 (36.4-41.3) | 55.3 | 5.9 |  | 1.38 (0.76-0.93) | 1.40 (1.32-1.49) | 39.2 (36.8-41.6) |
| South West | 1,343 | 23.6 (21.4-26.0) | 49.9 | 26.5 |  | 0.84 (0.76-0.93) | 1.19 (1.08-1.31) | 33.2 (30.0-36.4) |
|  |  |  |  |  |  |  |  |  |
| **Trimester of screening test** |  |  |  |  |  |  |  |  |
| First (11-13 weeks' gestation) | 304,539 | 33.0 (32.8-33.1) | 58.5 | 8.5 |  | 1.00 (ref) | 1.00 (ref) | 33.4 (33.2-33.6) |
| Second (15-20 weeks' gestation) | 162,321 | 25.4 (25.2-25.6) | 55.3 | 19.3 |  | 0.77 (0.76-0.78) | 0.73 (0.72-0.74) | 24.4 (24.2-24.7) |

All p-values for relative risks are <0.001

*Adjusted for all other factors
